# Predictors and occurrence of postoperative cognitive dysfunction in children undergoing noncardiac surgery: A prospective cohort study

**DOI:** 10.1002/ibra.12066

**Published:** 2022-09-10

**Authors:** Fang‐Fang Han, Xiu‐Mei Wang, Hai‐Jun Zhang, Jun‐Ze Wang, Zhen‐Xing Bao, Yu‐Lan Li

**Affiliations:** ^1^ The First Clinical Medical College of Lanzhou University Lanzhou Gansu P.R. China; ^2^ Department of Anesthesiology and Operating Theater The First Hospital of Lanzhou University Lanzhou Gansu P.R. China

**Keywords:** anesthesia, children, cognitive dysfunction, surgery, surgical history

## Abstract

In children after cardiac surgery, alterations in cognitive ability and behavior are increasingly common, but whether postoperative cognitive dysfunction (POCD) occurs in children undergoing noncardiac surgery is not known. The present study was performed to investigate the incidence rate and potential risk factors of early neurocognitive dysfunction in children after noncardiac surgery. Two hundred patients aged between 4 and 14 years old underwent elective noncardiac surgery and 100 healthy age‐matched controls were enrolled in this prospective observational study. Wechsler Preschool and Primary Scale of Intelligence or Wechsler Intelligence Scale for Children‐Revised were conducted 1 day before and 3 days after surgery. POCD was calculated and diagnosed as a combined *Z* score. Any factors that differed between POCD and non‐POCD group (*p* < 0.10) were tested together by multivariate logistic regression analysis against the cognitive outcome of patients, to find out the independent risk factors of POCD. The general incidence of POCD was 15.6%. The univariate analysis revealed that POCD was associated with general anesthesia, surgical and anesthesia duration, early postoperative fever (EPF), and surgical history. However, only the history of surgery (*p* = 0.029), anesthesia duration (*p* = 0.010), and EPF (*p* < 0.001) were demonstrated to be independent risk factors for POCD. The occurrence rate of early POCD after noncardiac surgery in children is 15.6%. Children who had surgical history, longer anesthesia duration, or EPF are more prone to develop POCD.

## Graphical abstract

Postoperative cognitive dysfunction (POCD) occurs in children undergoing cardiac surgery and is increasingly common. This study was performed to investigate the incidence rate and potential risk factors of early neurocognitive dysfunction in children after noncardiac surgery. This studyfound that the general incidence of POCD was 15.6%. POCD was associated with general anesthesia, surgical and anesthesia duration, early postoperative fever (EPF), and surgical history. Only the history of surgery, anesthesia duration, and EPF were demonstrated to be independent risk factors for POCD. It means that children who had surgical history, longer anesthesia duration, and EPF were prone to have POCD after noncardiac surgery. WPPSI, Wechsler Preschool and Primary Scale of Intelligence. WISC‐R, Wechsler Intelligence Scale for Children‐Revised.

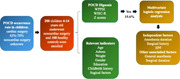

## 1 INTRODUCTION

Memory loss and lack of concentration are symptoms that frequently occur in patients undergoing surgical procedures. Some patients complained that they were “just not the same” even if they underwent their surgery several years previously. Symptoms of subtle cognitive decline after surgery are usually described as “postoperative cognitive dysfunction (POCD),” characterized by impairment of memory, attention, and concentration detected by neuropsychological testing.^1^

Research evaluating cognitive decline after surgery has primarily focused on elder patients, who might have an increased vulnerability to neurological deterioration, with the incidence rate of POCD reported ranging from 6.6% to 67%.^2^, ^3^ The etiology of postoperative cognitive deficits is multifactorial, with known risk factors including increased age, lower educational level, duration of surgery, previous surgical procedure, and postoperative complications.^4^, ^5^

Detecting POCD in children whose brain is developing is also important. Several studies reported alterations in cognitive abilities and behavior in young children after surgery and anesthesia, such as attention seeking, crying, temper tantrums, sleep disturbance, anxiety, and even neurodevelopmental impairment, especially in children who underwent congenital cardiac surgery associated with cardiopulmonary bypass (CPB).^6^, ^7^, ^8^ But less is known about the prevalence of POCD after noncardiac surgery.

The aim of this study was to characterize the incidence of POCD in children after noncardiac surgery and find out the clinical predictors for these abnormalities.

## 2 METHODS

### 2.1 Ethics

This prospective cohort study was approved by The First Hospital of Lanzhou University ethics committee in 2012 (LDYYLL2012‐0005) and registered as a clinical trial by the Chinese Clinical Trial Registry in 2012 (ChiCTR‐RCS‐12003072). Written informed consent was obtained from all subjects or their parents or guardians before enrollment.

### 2.2 Subjects

A total of 200 patients aged between 4 and 14 years old undergoing elective noncardiac surgery were recruited in this study. Surgery procedures were mainly ear nose throat (tonsillectomy, adenoidectomy, etc.), and general surgery (mainly laparoscopic hernia sac high ligation). Exclusion criteria were as follows: central nervous system disease, congenital disease, attention deficit hyperactivity disorder, severe visual or auditory disorders, and unwillingness to comply with the protocol or procedures. During the study period, 100 age‐matched controls that did not undergo surgery or anesthesia were enrolled randomly according to their school registration numbers, and all their physical examinations were confirmed healthy.

Information about the patients′ demographic status, physical examination, and current medications was recorded. The childbirth history (children delivered vaginally or via cesarean delivery), surgical history (previous surgical procedure with anesthesia), education duration (started from junior kindergarten which was recorded as 1‐year education), and address representing living environment (children lived in the city or in the village) were also recorded.

### 2.3 Treatment

The anesthesia methods used in this study were as follows: general anesthesia with endotracheal intubation and caudal epidural block with intravenous propofol (1−2 mg kg^−1^) sedation, with the aim of maintaining a bispectral index < 60 (BIS Monitor; Philips intellivue, MP50).

As premedication exacerbates the impairment of children΄s cognition after anesthesia, thus obscuring the effects of the latter, no child received premedication. General anesthesia was induced with midazolam 0.04−0.05 mg kg^−1^, fentanyl 1−2 μg kg^−1^, rocuronium 0.5−1.0 mg kg^−1,^ and propofol 1−2 mg kg^−1^. Intubation was done after induction administration for over 1 min. Ventilation support was given immediately after intubation with oxygen. Anesthesia was maintained by intravenous anesthesia with propofol or volatile anesthetics (isoflurane or sevoflurane). A mixture of 1% lidocaine and 0.25% bupivacaine 1−1.5 mg kg^−1^ was used in the caudal epidural block.

Pulse oximetry, noninvasive blood pressure, end‐tidal carbon dioxide partial pressure, and five‐channel electrocardiography were monitored. Systemic analgesia was with conventional pediatric doses of diclofenac, ibuprofen, and paracetamol. Opioids were not routinely used as the known effects of opioids upon cognitive function would obscure any effects due to the anesthetic. Nonsteroidal analgesics were given when necessary. Duration of anesthesia and surgery, postoperative complications, and any accident that happened during the operation were recorded.

### 2.4 Cognitive assessment

All the neuropsychological tests were carried out in a quiet room with the patient and investigator who was blinded to the patients′ conditions. If the children could not be tested in the test room, a quiet setting in the ward would be found for the test. Parental presence was allowed with younger children who were difficult to coordinate, and this could not affect the performance scores because they were not allowed to answer any questions.

The preoperative evaluation was administered the day before surgery, and the postoperative testing session was performed 3 days (d) after surgery at around the same time of the day to avoid changes in cognitive function caused by time. Patients who were too unwilling to take the postoperative tests were administrated as soon as possible thereafter. All controls were tested with the same interval and the same investigator.

A battery of six neuropsychological tests primarily focused on language, comprehension, and memory was chosen from the Wechsler Preschool and Primary Scale of Intelligence (WPPSI) and Wechsler Intelligence Scale for Children‐Revised (WISC‐R) to examine cognitive function.^9^ All following tests were fulfilled within 40 min.

To keep the scores comparable among tests, the primitive scores of each test were converted to scale scores ranges from 1 to 19 for further analysis according to children′s exact age and Wechsler′s form.

### 2.5 POCD definition

The extent of POCD was calculated using the combined *Z* score^10^ described as follows: For controls, the changes of two times scores for each test from pre‐ to postoperative testing session were compared. The mean ± SD of these differences was calculated. For patients, the changes from preoperative scores to postoperative ones were also compared. Then, these changes were used to subtract the mean changes of the control group, and the result was divided by the standard deviation of the control group to obtain a *Z* score for each test. The positive *Z* scores indicated deterioration in cognitive function from the preoperative test. A composite *Z* score was calculated as the sum of the *Z* scores for an individual patient divided by the standard deviation for the sum of the *Z* scores of the control group.

POCD was diagnosed when the *Z* scores in two individual tests or the composite *Z* score was 1.96 or greater. This definition identifies patients with general deterioration in all tests or substantial deterioration in some fields of cognition.

### 2.6 Statistical analysis

Data were analyzed using the SPSS software package, version 13.0 for Windows (SPSS Inc.). The comparison of neuropsychological test scores before and after surgery was analyzed by the paired two‐tailed Student's *t* tests. Meanwhile, comparisons of continuous variables between groups were accomplished with the independent Student's *t‐tests*. The comparisons of proportions between groups were accomplished with the *χ* ^2^ test or Fisher′s exact test.

Patients were divided into two groups according to whether POCD occurred or not, namely the POCD group (POCD) and the non‐POCD group (non‐POCD). Associated factors of POCD were analyzed using the independent Student's *t* tests. Factors that differed between POCD and non‐POCD groups (*p* < 0.10) were tested together by multivariate logistic regression analysis against the cognitive outcome of patients, to find out the independent risk factors of POCD.

In the model of multivariate logistic regression analysis, we chose *Y* = 0 with respect to non‐POCD and *Y* = 1 respect with to POCD in the dependent variable group. The independent variables were as follows: *X*1, age; *X*2, anesthesia duration; *X*3, surgical duration; *X*4 = 1, children delivered vaginally; *X*4 = 2, children delivered via Cesarean delivery; *X*5 = 0, children without surgical history; *X*5 = 1, children with surgical history; *X*6 = 0, children without early postoperative fever (EPF); *X*6 = 1, children with EPF.

Odds ratios (ORs) and regression coefficients with 95% confidence intervals (CIs) were determined in the logistic regression analysis. Two‐tailed *p* < 0.05 was considered to be statistically significant.

## 3 RESULTS

### 3.1 Demographic information

**Figure 1:**
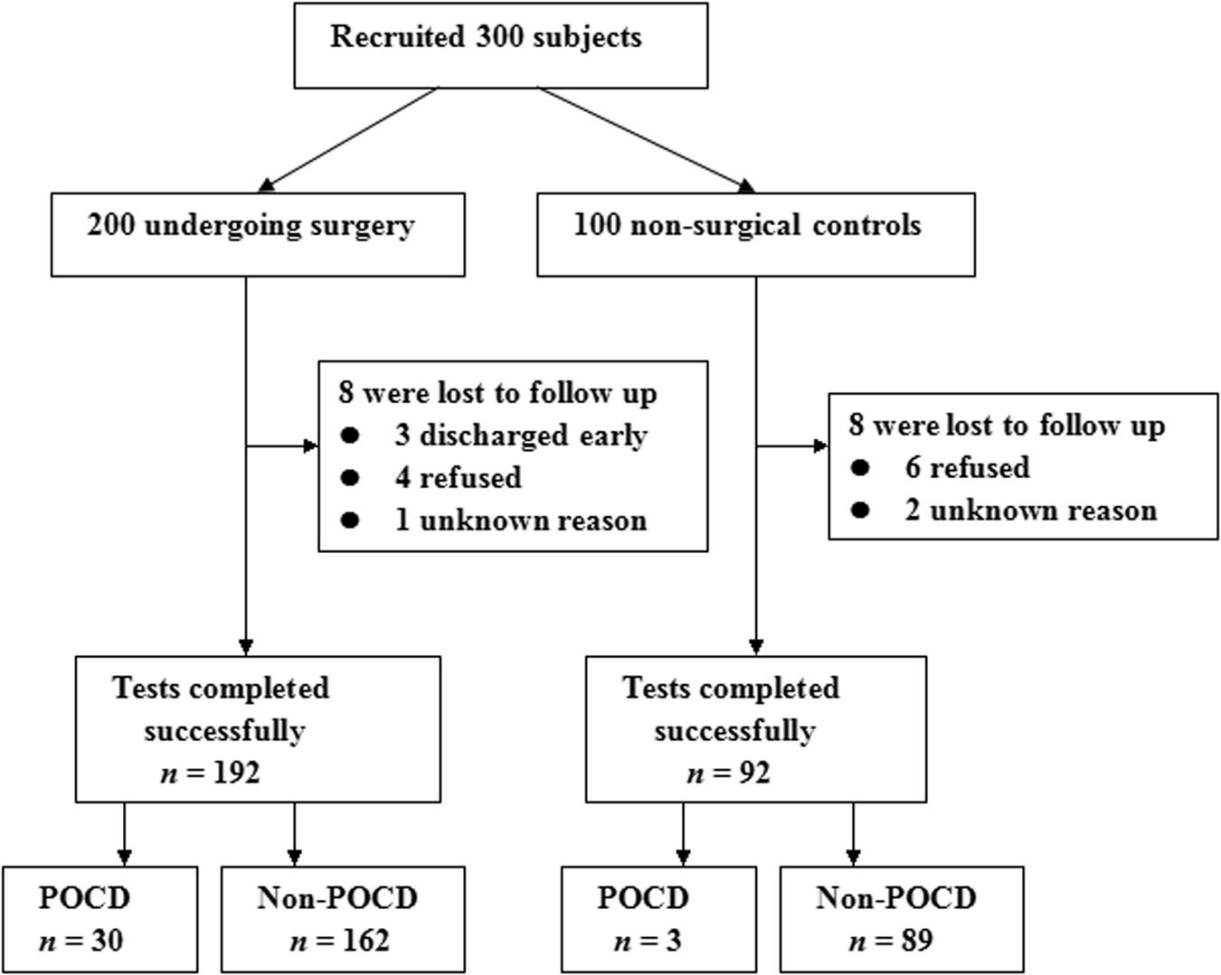
Flowchart of the subject recruitment process and study design. POCD, postoperative cognitive dysfunction.

The study recruited 200 noncardiac surgical patients, 8 of whom did not complete the postoperative assessment. During the same period, none of the controls was hospitalized, and eight dropped out. The main reason for the loss of follow‐up was children's refusal. The final analysis included 192 surgical patients and 92 controls (Figure 1). There was no significant difference between groups in baseline characteristics (Table 1). EPF (>38.0°C) occurred in 30 patients, and all were cured when they were discharged from the hospital (Table 3).

**Table 1:** Participant demographic of controls and patients

	Controls (*n* = 92)	Patients (*n* = 192)
Age (years)	8.6 ± 2.7	8.6 ± 2.8
Weight (kg)	29.4 ± 8.7	30.4 ± 12.6
Education (years)	5.6 ± 2.5	5.5 ± 2.6
Gender		
Male	59 (64.1%)	134 (69.8%)
Female	33 (35.9%)	58 (30.2%)
Childbirth		
DV	61 (66.3%)	133 (69.3%)
CD	31 (33.7%)	59 (30.7%)
Address		
City	48 (52.2%)	89 (46.4%)
Village	44 (47.8%)	103 (53.6%)

*Note*: Data are mean ± SD or *n* %. No significant differences were observed between groups for any variable.

Abbreviations: CD, children delivered via Cesarean delivery; DV, children delivered vaginally.

### 3.2 Cognitive outcome

The scores for all six neuropsychological tests at baseline and 3 days after surgery are presented in Table 2. Compared with the controls, there were significantly differences from patients in their scores of information (*p* = 0.002), arithmetic (*p* = 0.005), comprehension (*p* < 0.001), and coding (*p* = 0.004) at later testing sessions.

**Table 2:** Neuropsychological results for patients (*n* = 192) and controls (*n* = 92) at baseline and 3 days after surgery

Test	Group	Preoperative score	*p* *	Postoperative score	*p* **	*p* ***
Information	Controls	9.5 ± 2.1	0.002	9.4 ± 2.1	0.002	0.384
	Patients	8.5 ± 3.3		8.4 ± 3.1		
Vocabulary	Controls	9.7 ± 2.2	0.499	9.9 ± 2.2	0.293	0.000
	Patients	10.0 ± 3.5		9.6 ± 3.2		
Arithmetic	Controls	9.0 ± 1.9	0.658	9.1 ± 2.0	0.005	0.000
	Patients	8.8 ± 3.6		8.2 ± 3.4		
Comprehension	Controls	10.0 ± 2.0	0.018	10.3 ± 2.0	0.000	0.003
	Patients	9.3 ± 2.8		9.0 ± 2.7		
Digit span	Controls	8.7 ± 2.2	0.377	9.0 ± 2.2	0.638	0.213
	Patients	9.0 ± 2.8		8.9 ± 2.8		
Coding	Controls	9.4 ± 2.5	0.166	9.9 ± 2.8	0.004	0.550
	Patients	8.9 ± 2.8		8.8 ± 3.2		

*Note*: Data are mean ± SD. p* = comparison between groups preoperatively; p** = comparison between groups postoperatively; p*** = comparison between pre‐ and postoperative scores in patients.

At 3 days postoperatively, the general incidence of early POCD was 15.6% (30/192) in noncardiac surgical patients, and 3 of 92 controls (3.3%) experienced a cognitive decline (*p* < 0.001). There was no statistically significant difference in the incidence of POCD in children undergoing different noncardiac surgeries (*p* = 0.844) (Table 3). Surgical types include ENT surgery and general surgery (mainly laparoscopic hernia sac high ligation).

**Table 3:** Perioperative factors and the presence of POCD in patients

	Non‐POCD (*n* = 162)	POCD (*n* = 30)	*p* *
Age (years)	8.6 ± 2.7	9.0 ± 3.1	0.427
Weight (kg)	30.7 ± 12.6	32.3 ± 13.4	0.378
Education (years)	5.4 ± 2.6	5.8 ± 2.7	0.427
Gender			
Male	116 (71.6%)	18 (60.0%)	0.203
Female	46 (28.4%)	12 (40.0%)	
Childbirth			
DV	117(72.2%)	16 (53.3%)	0.052
CD	45 (27.8%)	14 (46.7%)	
Address			
City	77 (47.5%)	12 (40.0%)	0.551
Village	85 (52.5%)	18 (60.0%)	
Surgical type			
ENT surgery	80 (49.4%)	14 (46.7%)	0.844
General surgery	82 (50.6%)	16 (53.3%)	
Surgical duration (min)	52.6 ± 31.6	79.3 ± 54.3	0.013
Anesthesia duration (min)	77.3 ± 35.7	112.0 ± 57.2	0.003
Anesthesia type			
GA	90 (55.6%)	30 (100%)	0.000
CAB	72 (44.4%)	0	
Early postoperative fever	4 (2.5%)	8 (26.7%)	0.000
Surgical history	30 (18.5%)	16 (53.3%)	0.000

*Note*: Data are mean ± SD or *n* %. p* = comparison between groups.

Abbreviations: CAB, caudal epidural block with propofol sedation; CD, children delivered via Cesarean delivery; DV, children delivered vaginally; ENT, ear nose throat; GA, general anesthesia; POCD, postoperative cognitive dysfunction.

### 3.3 Univariate analysis

Univariate analysis was performed to find any potential risk factors of POCD (Table 3). It revealed that general anesthesia (*p* < 0.001), duration of anesthesia (*p* = 0.003) and operation (*p* = 0.013), EPF (*p* < 0.001), and surgical history (*p* < 0.001) were associated with POCD.

### 3.4 Multivariate analysis

**Figure 2:**
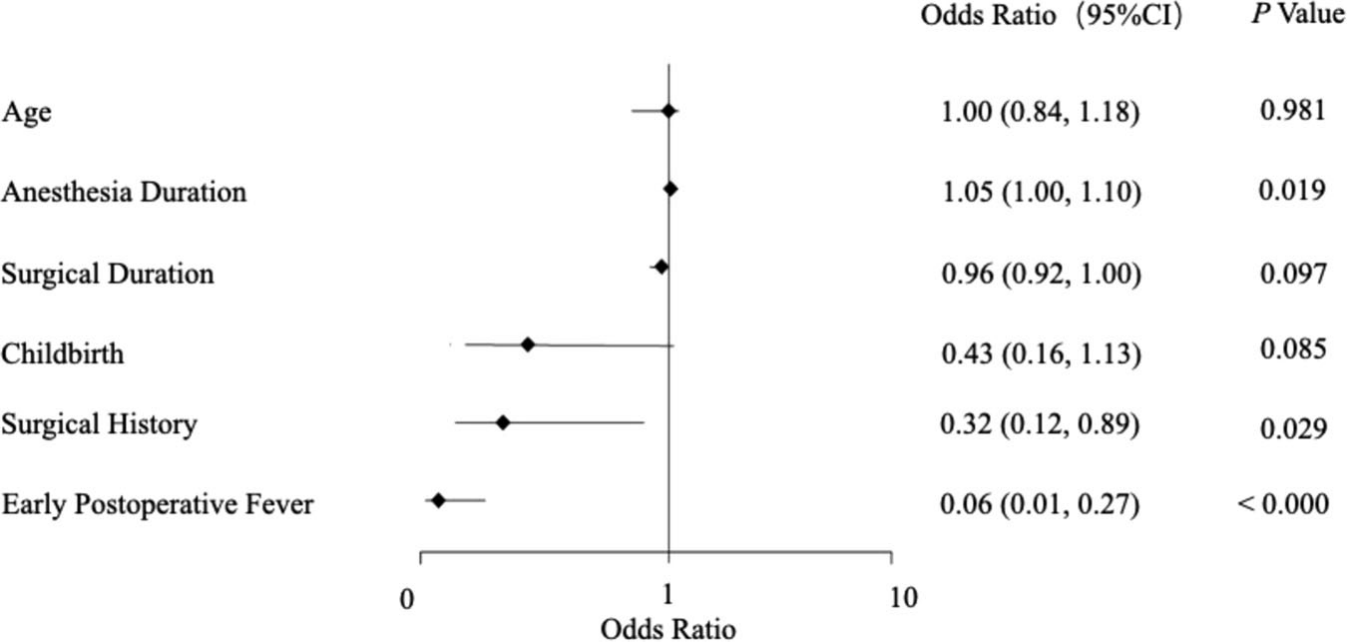
Multivariate logistic regression analysis of risk factors for POCD in patients (*n*= 192). POCD was assessed 3 days postoperatively. Data presented as odds ratios with 95% confidence intervals. POCD, postoperative cognitive dysfunction.

The factors with a *p* value lower than 0.1 in the univariate analysis were screened for multivariate analysis, including age, duration of anesthesia and operation, EPF, surgical history, and childbirth history. Among these factors, continuous measurement data were converted to discrete data and then all the factors including additional children's age and childbirth history were admitted into logistic regression analysis. Finally, the independent risk factor for POCD was the history of surgery (*p* = 0.029), anesthesia duration (*p* = 0.010), and EPF (*p* < 0.000) (Figure 2).

## 4 DISCUSSION

The incidence rate of POCD in children after noncardiac surgery was 15.6%, and it was associated with general anesthesia, surgical duration, anesthesia duration, EPF, and surgical history. The history of surgery, anesthesia duration, and EPF was proved to be independent predictors of POCD.

To our knowledge, this is one of the few investigations of POCD in children after noncardiac surgery. We found a lower incidence of early POCD of 15.6% in this age group thanprevious studies in children after cardiac surgery (42%−70%) with perhaps the result of organic cerebral damage associated with CPB,^11^ but we still demonstrated that children were likely to develop POCD at early period after noncardiac surgery.

The scale used in this study is WPPSI and WISC‐R specially designed for children, which can indirectly reflect children's cognitive abilities such as emotions, perceptions, etc. In our results, the most frequent postoperative neuropsychological performance decline (*p* ≤ 0.01) of children after surgery occurred in the Vocabulary and Arithmetic tests, which are designed to examine the function of verbal learning and mental attention. This is similar to previous studies.^7^, ^12^, ^13^ The changes in cognitive function after cardiac surgery, including the reduction of comprehensive abilities such as attention and mathematical calculation ability, may have long‐term effects. Cognitive changes also occur after noncardiac surgery in children.^14^

Our study shows that children who underwent general anesthesia were at increased risk of developing POCD, and the duration of anesthesia is also an independent factor of POCD. About this point, some studies found significant relationships^15^, ^16^, ^17^, ^18^ whereas some others found no correlation.^19^ Previous studies have shown that POCD‐associated general anesthesia was related to systemic inflammatory response,^20^ oxidative stress,^21^ and neuroinflammation^22^ caused by surgical trauma. Recent studies have also found that general anesthesia can also mediate changes in gut microbiota composition that cause POCD development.^23^

In our hospital, children's surgery requires the removal of the tracheal tube after general anesthesia before being sent to the Post Anesthesia Care Unit. Therefore, there is a time difference between the duration of surgery and anesthesia. A longer duration of anesthesia also means more types of drugs and more doses. The combinational and prolonged use of different anesthetics on the vulnerable brain during general anesthesia may exacerbate the neurotoxic effect and lead to the decline of cognitive function.

The results of the present study suggest that the history of surgery is one independent risk factor for POCD. Sun^24^ showed that receiving anesthesia and surgery at children's early age might be associated with later neurocognitive development, especially at a vulnerable age and receiving multiple anesthetics. While Hansen et al.^25^ found that a single, relatively brief anesthetic exposure with hernia repair in infancy did not reduce academic performance at 15 or 16 years old, some surgical children were developmentally disadvantaged compared with the background population in that study. However, the history of repeated surgeries in childhood still has an impact on children's postoperative cognitive function.^7^, ^26^

EPF is another independent risk factor of POCD. Postoperative fever is a common complication of cardiac operations using CPB. The frequency of postoperative fever in cardiovascular surgery varies from 12% to 73% in studies of adults and children.^27^ EPF is common following all types of operations, occurring in anywhere from 25% to 75% of adult patients depending on the definition used.^28^ A variety of noninfectious factors commonly contribute to fever in these postoperative patients, including the normal inflammatory cytokine response to surgery, perioperative medications, hematoma reabsorption, and the tissue trauma associated with surgery. EPF reflects an inflammatory response initiated either in response to the surgical trauma itself or to the interaction of blood with the foreign surfaces of the CPB circuit, which also represents a risk factor for developing POCD in children.^28^, ^29^

EPF is also common in the elective pediatric surgical population and is rarely associated with positive blood, urine, respiratory cultures, and/or chest radiograph suggestive of an infectious source.^28^ In this study, no pediatric patients had significant positive changes in blood routine examinations and blood bacterial cultures, that is, there was no clear indication for postoperative infection. All patients recovered spontaneously upon discharge.

Generally, the proinflammatory cytokines always lead to a systemic acute‐phase response, which may include a wide variety of behavioral, physiological, and psychological changes in the early period after surgery. But the fever and discomfort associated with EPF may somewhat distract the children and lead to poorer performance in the tests. Obviously, EPF and inflammation have enlarged the effects, and influence cognitive ability indirectly.

As Monk et al.^10^ suggested that a major limitation of studies investigating POCD is the differences in research methodologies and statistical methods. The methodology of combined *Z* scores we used to define POCD is recommended by the ISPOCD1 study,^5^ and showed clear differences in substantial deterioration in special tests (e.g., verbal learning and attention) to warrant further exploration.

Traditionally, the age of 3 years was considered to be the “window of vulnerability” in humans, and neurocognitive development is rapid in the 5−12‐year age range.^18^ So the children between 4 and 14 years were involved because we think most of the vulnerable age period of the developing brain was included, but we cannot get the cognition scores of children younger than 4 years old because they are too difficult to cooperate with.

The absence of long‐term follow‐up testing is another limitation of this study. Because the types of surgery included were mainly short surgeries with a short hospital stay of 3−5 days which included orchidopexy, appendectomy, nasal cystectomy, ENT, and other noncardiac surgeries. In addition, it is difficult to unify the test personnel and the venue after discharge. Further research should pay more attention to the long‐term effects of anesthesia and surgery on children's cognitive function.

In conclusion, the incidence of early POCD in noncardiac surgical children was 15.6%, and it was not influenced by age, education duration, and childbirth history. POCD was associated with general anesthesia, surgical and anesthesia duration, EPF, and surgical history. The history of surgery, anesthesia duration, and EPF were proved to be independent predictors for the development of cognitive dysfunction in noncardiac surgical children.

## AUTHOR CONTRIBUTIONS

Fang‐Fang Han conceived the study and its design, carried out the experiments, and approved the final manuscript. Yu‐Lan Li participated in its design and coordination and helped to revise the manuscript. Xiu‐Mei Wang analyzed the original data reported in this manuscript. Hai‐Jun Zhang helped to perform the experiments and collected data. Jun‐Ze Wang and Zhen‐Xing Bao rechecked, organized, and analyzed the original data again.

## CONFLICT OF INTEREST

The authors declare no conflict of interest.

## ETHICS STATEMENT

The study was approved by the ethics committee of the First Hospital of Lanzhou University (LDYYLL2012‐0005) and registered as a clinical trial by the Chinese Clinical Trial Registry (ChiCTR‐RCS‐12003072).

## Data Availability

The data that support the findings of this study are available from the corresponding author upon reasonable request. The data are not publicly available due to privacy or ethical restrictions.
